# Cost-effectiveness of telehealth for patients with raised cardiovascular disease risk: evidence from the Healthlines randomised controlled trial

**DOI:** 10.1136/bmjopen-2016-012352

**Published:** 2016-08-26

**Authors:** Padraig Dixon, Sandra Hollinghurst, Louisa Edwards, Clare Thomas, Daisy Gaunt, Alexis Foster, Shirley Large, Alan A Montgomery, Chris Salisbury

**Affiliations:** 1 Centre for Academic Primary Care, School of Social and Community Medicine, University of Bristol, Bristol, UK; 2 Bristol Randomised Trials Collaboration (BRTC), School of Social and Community Medicine, University of Bristol, Bristol, UK; 3 Clinical Trials Research Unit, School of Health and Related Research (ScHARR), University of Sheffield, Sheffield, UK; 4 Public Health England, Hampshire, UK; 5 Nottingham Clinical Trials Unit, Faculty of Medicine & Health Sciences, University of Nottingham, Nottingham, UK

**Keywords:** HEALTH ECONOMICS, STROKE MEDICINE, Angina

## Abstract

**Objectives:**

To investigate the cost-effectiveness of a telehealth intervention for primary care patients with raised cardiovascular disease (CVD) risk.

**Design:**

A prospective within-trial patient-level economic evaluation conducted alongside a randomised controlled trial.

**Setting:**

Patients recruited through primary care, and intervention delivered via telehealth service.

**Participants:**

Adults with a 10-year CVD risk ≥20%, as measured by the QRISK2 algorithm, with at least 1 modifiable risk factor.

**Intervention:**

A series of up to 13 scripted, theory-led telehealth encounters with healthcare advisors, who supported participants to make behaviour change, use online resources, optimise medication and improve adherence. Participants in the control arm received usual care.

**Primary and secondary outcome measures:**

Cost-effectiveness measured by net monetary benefit at the end of 12 months of follow-up, calculated from incremental cost and incremental quality-adjusted life years (QALYs). Productivity impacts, participant out-of-pocket expenditure and the clinical outcome were presented in a cost-consequences framework.

**Results:**

641 participants were randomised—325 to receive the telehealth intervention in addition to usual care and 316 to receive only usual care. 18% of participants had missing data on either costs, utilities or both. Multiple imputation was used for the base case results. The intervention was associated with incremental mean per-patient National Health Service (NHS) costs of £138 (95% CI 66 to 211) and an incremental QALY gain of 0.012 (95% CI −0.001 to 0.026). The incremental cost-effectiveness ratio was £10 859. Net monetary benefit at a cost-effectiveness threshold of £20 000 per QALY was £116 (95% CI −58 to 291), and the probability that the intervention was cost-effective at this threshold value was 0.77. Similar results were obtained from a complete case analysis.

**Conclusions:**

There is evidence to suggest that the Healthlines telehealth intervention was likely to be cost-effective at a threshold of £20 000 per QALY.

**Trial registration number:**

ISRCTN27508731; Results. Prospectively registered 05 July 2012.

**Strengths and limitations of this study**

- We report a within-trial economic evaluation of the one of the largest randomised controlled trials designed to evaluate a telehealth-based complex intervention for the management of cardiovascular disease risk.
- This prospective economic evaluation used detailed patient-level data to contribute to the small body of evidence concerned with the cost-effectiveness of telehealth for patients with long-term conditions.
- The intervention was likely to be cost-effective when adopting a UK health system perspective, a conclusion that held whether complete case or imputed data were analysed.
- The recruitment rate to the trial was relatively low, and this may affect the generalisability of these findings.

## Introduction

Cardiovascular disease (CVD) is the leading cause of death worldwide.^1^ Many of these deaths could be avoided by addressing modifiable behavioural risk factors such as smoking, diet and exercise.^1–4^ Costs associated with CVD are substantial,^5^ ^6^ and predicted to increase.^7^ ^8^

There is an urgent need to identify cost-effective healthcare interventions that can effect behavioural changes, address the burden of CVD, and efficiently and effectively support patient care. The Healthlines study was a multicentre, parallel two-arm and individually randomised controlled trial (RCT) designed to assess the effectiveness and cost-effectiveness of a telehealth intervention for primary care patients. The intervention was intended to promote behaviour change, optimisation of medication, improved coordination of care and improved medication adherence in patients with a high risk of developing CVD. Development of the intervention,^9^ the protocol for the trial^10^ and the main results of the trial have been published elsewhere.^11^

Evidence of the effectiveness and cost-effectiveness of telehealth in general,^12^ and for the management of patients with elevated CVD risk,^13^ is mixed. In this paper, we report the results of an economic evaluation conducted alongside the Healthlines RCT. We estimated the cost-effectiveness of the telehealth service from a National Health Service (NHS) perspective for primary care patients with elevated CVD risk. A companion paper^14^ complements the within-trial evaluation presented in this paper with simulation modelling of the cost-effectiveness of this intervention over the remaining lifetime of trial participants.

## Methods

### RCT setting and participants

Participants aged between 40 and 74 on the date of invitation were recruited from 42 general practices in or near Bristol, Sheffield and Southampton. Participants were individuals with a 10-year risk of a cardiovascular event of ≥20% calculated using the QRISK2 algorithm,^15^ and with at least one of the following modifiable CVD risk factors: (1) systolic blood pressure ≥140 mm Hg, (2) body mass index (BMI) ≥30 kg/m^2^ and/or (3) a current smoker.

Individuals were excluded from the trial if they had a confirmed diagnosis of CVD (defined as history of heart attack, angina, heart failure, stroke or transient ischaemic attack), currently or planning to be pregnant, or unable to communicate verbally in English. In total, 641 participants were randomised on a 1:1 basis to receive either the telehealth intervention in addition to usual care or usual care alone for up to 12 months.

An NHS perspective was adopted for the cost-effectiveness analysis, which compared NHS costs with quality-adjusted life years (QALYs) over the 12 months of trial follow-up. Self-reported data on personal expenditure and productivity impacts were also collected and are presented as part of a cost-consequences analysis.

### Intervention

A structured programme of work (C Salisbury, A O'Cathain, C Thomas, *et al.* Telehealth for patients with long-term health conditions: development and evaluation of the Healthlines Service. NIHR Journals Library—Programme Grants for Applied Research, Under review) was used to develop the de novo, theory-driven, web and phone-based telehealth service received by participants in the intervention arm. The core feature of the Healthlines Service consisted of scripted telephone support and responsive advice delivered by NHS Direct Health Information Advisors (HIAs). The scripts used in the telephone encounters, based on a successful US intervention,^16^ ^17^ focused on goal setting, stimulus control and problem solving to address modifiable risk factors for CVD. These encounters were responsive to need, and participants could, for example, request calls with supervisors, and could ask to be directed to sources of information relevant to the management of their condition. Participants were eligible to receive up to 13 scheduled telephone encounters delivered approximately every 4 weeks over the course of the 12 months of trial follow-up. The intervention was in addition to usual care.

Participants with systolic blood pressure ≥140 mm Hg and without atrial fibrillation were offered a home blood pressure monitor. Blood pressure was reviewed during each encounter with the Healthlines advisor and targets were set based on guidelines issued by the National Institute for Health and Care Excellence (NICE).^18^ General practitioners were advised if patients were not adherent to medication. Participants in the intervention arm were provided with access to an online web portal containing summaries of progress (such as graphs of blood pressure against target), and access to online resources relevant to the management of their condition.

Participants in the control arm received unmodified usual care.

### Measurement and valuation of outcomes

The primary outcome of the RCT was the proportion of participants responding to treatment, defined as a binary outcome reflecting maintenance or reduction of 10-year CVD risk estimated using the QRISK2 algorithm at 12 months after randomisation. QRISK2 scores at 12 months were calculated by updating age and modifiable risk factors, with all other variables held constant.

Participants responded to questions concerning health-related quality of life at baseline and at 6 and 12 months postrandomisation using the EQ-5D-5L measure.^19^ This generic instrument measures five dimensions (mobility, self-care, usual activities, pain and discomfort) of health-related quality of life, and uses five categories to characterise health states associated with these dimensions: no problems, slight problems, moderate problems, severe problems and extreme problems. The EQ-5D-5L UK valuations were not available to us at the time of trial analysis, and hence the EQ-5D-5L responses were ‘cross-walked’ to the three-level version of the instrument (EQ-5D-3L) and valued using the Euroqol value set for the UK.^20^

### Measurement and valuation of resource use

Relevant primary care consultations and information on prescriptions related to CVD risk (lipid regulating, antihypertensives, antiplatelet, obesity treatments and nicotine dependence medication) were collected (with consent) from medical records. Primary care consultations were costed using Curtis.^21^ Prescribed medications were costed using the Prescription Cost Analysis England (PCAE) database,^22^ and checked against the British National Formulary.^23^

Questionnaires issued to participants at 6 and 12 months were used to collect information on healthcare use associated with CVD risk not available from primary care records. Examples of these resources include hospital and ambulance use, district nurse consultations, and use of NHS walk-in centres. Since we imputed costs rather than resource use per se for our base case analysis, we present resource use for available and complete cases in the online supplementary material rather than in the main text.

10.1136/bmjopen-2016-012352.supp1 Supplementary data

The sources used to value these resources uses were primarily Curtis,^21^ NHS National Reference Costs for 2012/2013^24^ and other sources as described in the online supplementary tables A1 and A2. Participants also reported use of private health services (eg, private nutritionists), and out-of-pocket expenditure (eg, self-help books) associated with CVD risk. Participants reported time absent from work due to CVD risk, which was valued, where appropriate, at the national median gross hourly wage for 2013 of £11.59.^25^

The estimated cost of the intervention was based on the number and length of telephone calls, the number of failed attempts made by HIAs to contact participants, blood pressure monitors and the costs associated with establishing the service, such as training costs. Unit costs for these resources are described in the online supplementary table A3.

HIAs worked 40 hours per week, and were remunerated at band 4 of the NHS ‘Agenda for Change’ pay scale. Salaries and associated costs (such as overhead) were based on Curtis.^21^ The cost per hour of participant contact time was estimated based on a ratio of contact to non-contact time calculated from task-scheduling diaries. Initial and ongoing training of HIAs was provided by a nurse-grade trainer; the training was assumed to last for 3 years and costs were amortised on that basis. This assumes that training of this type would not be redundant after 1 year; instead, it assumes that the training would be effective for 3 years, after which additional training might become necessary.

Costs are reported in 2012/2013 pounds sterling.

### Analysis of data

Summary statistics were estimated for all variables used in the economic analysis, and any missingness was identified. All cost-effectiveness analysis was conducted on an ‘intention-to-treat’ basis. Neither costs nor benefits were discounted given the follow-up period of the trial.

A very small proportion of patients (<0.5%) had missing data on primary care consultations. This was primarily due to patients having moved during the study to different practices. Mean values were imputed for these patients. Complete cases refer to cases constructed after initial data cleaning and mean imputation of the small amount of missing primary care consultation data. These complete cases had no other missing cost or EQ-5D-5L data needed to undertake inferential cost-effectiveness analysis from the perspective of the NHS. Approximately 82% of participants were categorised as complete cases under this definition.

Multiple imputation was used for all other missing data. Aggregate costs at the level of primary care, medication, other NHS costs and responses to the EQ-5D-5L at each time point were imputed using the—ice—command^26^ ^27^ in Stata V.13.1, which implements multiple imputation by chained equations.

The imputation model included demographic, cost and clinical outcome variables at baseline and follow-up, stratified by trial arm. Predictive mean matching^27^ was applied to account for non-Gaussian distributions of some included variables, and passive imputation was used for those categorical variables that were functions of other included variables.

The number of imputations (n=60) was selected to be greater than the proportion of missing data (18%).^27^ We followed the approach set out in Faria *et al*^28^ in order to implement ‘Rubin's rules’, so that variation within and between the set of 60 imputed data sets was reflected in the analysis of cost-effectiveness.

Health-related quality of life utilities were measured at baseline, and 6 and 12 months using responses to EQ-5D-5L questionnaires. QALYs were calculated from these responses using the ‘area under the curve’ method,^29^ and were adjusted for baseline differences in EQ-5D-5L scores.^30^

The cost-effectiveness analysis used seemingly unrelated regression (SUR), for which we rely on the near normality of differences in sample means and differences in sample variances in large samples.^31^ ^32^ We implemented SUR using the—sureg—command in Stata. We regressed costs and QALYs on a binary variable indicating allocation to trial arm. For the QALY equation, we also included controlled for baseline imbalances in utility.^30^ No other covariates were included in these regressions.

Incremental cost-effectiveness ratios (ICERs), cost-effectiveness acceptability curves (CEACs), net monetary benefit estimates^33^ and CIs around net monetary benefit point estimates were calculated parametrically from regression output. Net monetary benefit was estimated at the NHS threshold values suggested by NICE of £20 000–£30 000 per QALY.^34^

The cost-consequences analysis used all available cases, which were identified on a variable-by-variable basis. These were defined as having complete data across all relevant time points for any individual variable.

Stata V.13.1 software (Statacorp: College Station, Texas, USA) was used in all analyses.

### Sensitivity analysis

We carried out sensitivity analyses in two areas of uncertainty to test the robustness of our results. First, complete case analysis was conducted as a check on the base case imputed cost-effectiveness analysis. Second, the base case (imputed) results were assessed for their sensitivity to self-reported use of secondary care in order to assess the effect of rare but expensive events and to address potential recall bias or misclassification of resource use.

## Results

The trial recruited a total of 641 participants: 325 were randomised to receive the intervention and 316 received usual care in the control arm. The mean age of participants in the trial was 67.2 years, of whom 80% were male, and 99% of all participants were of white ethnicity. Mean QRISK2 score at baseline in the usual care arm was 30.8 (SD 9.5), and 31.1 (SD 10.2) in the intervention arm.

Compliance with the intervention was reasonably high; 8% of participants received two or fewer encounters with the HIAs (including the initial introductory encounter), 60% received 3–11 encounters and 32% received 12–13 encounters. The median number of encounters received was 10 (IQR 8–12). Two participants had missing data on the number of encounters received.

### Outcomes

There was an imbalance in the baseline imputed EQ-5D-5L scores between the two groups (0.77 (SE 0.01) in the control group vs 0.80 (SE 0.1) in the intervention group). Once this was adjusted for the size of the QALY gain was 0.012 in the imputed data set used for the base case analysis (table 1). Response to treatment, measured as the maintenance or reduction of QRISK2 scores, was modestly higher in the intervention arm (adjusted OR 1.3, 95% CI 1.0 to 1.9).

**Table 1:** Imputed QALYs

Cost and outcomes	Usual care (n=316*) mean (SE)	Intervention (n=325*) mean (SE)
Imputed unadjusted QALYs	0.774 (0.100)	0.810 (0.009)
Imputed QALYs, adjusted for baseline imbalance	0.786 (0.005)	0.798 (0.005)

*This sample size is based on 60 imputed data sets.

QALYs, quality-adjusted life years; SE, standard error.

### Resource use and cost

The mean cost per participant of providing the intervention was estimated to be £129 (SD £56.33), of which the cost of the encounter calls constituted 85% of total cost (table 2). NHS costs (primary care consultations, medication costs, use of NHS community services and NHS secondary care) were similar in each arm (table 3). Online supplementary tables A4–A10 provide disaggregated data on resource use and costs for available and complete cases (as defined above).

**Table 2:** Mean (SD) intervention cost (£) per participant for all participants and complete cases

Intervention elements	All participants (n=325) mean £ (SD)	Complete cases (n=262) mean £ (SD)
Encounter calls	108.80 (49.75)	114.68 (46.07)
Non-scheduled calls	1.39 (2.57)	1.47 (2.65)
All calls	110.20 (50.13)	116.15 (46.24)
Blood pressure monitor	18.92 (18.78)	18.89 (18.79)
Total cost per participant	129.12 (56.33)	135.04 (53.02)

**Table 3:** Imputed NHS costs

Cost and outcomes	N*	Usual care mean £ (standard error)†	Intervention mean £ (standard error)†
Imputed hospital, ambulance and other mean NHS costs	641	56 (19)	65 (22)
Imputed mean drug costs	641	67 (8)	67 (6)
Imputed mean primary care costs	641	241 (11)	242 (9)
Imputed mean NHS costs, excluding cost of the intervention	641	364 (26)	373 (26)
Imputed mean intervention costs	641	–	129 (3)
Imputed mean NHS-related costs, including cost of the intervention	641	364 (26)	502 (27)

*This sample size is based on 60 imputed datasets.

†Standard errors are reported for imputed data, rather standard deviations.

### Cost-consequences

Table 4 presents a cost-consequences matrix showing costs from different perspectives and a range of outcomes.

**Table 4:** Cost-consequence matrix

Cost and outcomes	Usual care	N	Intervention	N	Difference (95% CI)
Available data on costs (£)					
Mean cost of intervention	0	316	129	325	–
Mean cost of NHS resources, excluding intervention costs	361	283	362	285	1 (−72 to 76)*
Mean cost of NHS resources, including intervention costs	361	283	494	283	132 (57 to 212)*
Out-of-pocket expenses	64	298	79	299	15 (−20 to 50)*
Private healthcare	110	298	59	299	−50 (−141 to 1)*
Mean societal value per patient of lost production	76	298	52	299	−24 (−133 to 54)*
Consequences†					
QRISK2 response to treatment (proportion of responders)	43%	291	50%	295	Adjusted OR 1.3 (1.0 to 1.9)
EQ-5D-5L at 12 months, unadjusted for baseline‡	0.776	297	0.812	295	0.037 (0.007 to 0.070)*
QALYs, adjusted for baseline‡	0.788	279	0.799	275	0.01 (−0.014 to 0.040)*

*CI calculated as accelerated and bias corrected interval from 1000 bootstrap replicates to account for skewed distributions.

†All consequences measured at 12 months, or over a period of 12 months.

‡Based on available data.

NHS, National Health Service; QALYs, quality-adjusted life years.

Costs to the NHS were higher in the intervention arm, largely due to the cost of the intervention. The societal value of lost production was slightly higher per patient in the control arm, but not significantly so. The comparison is affected by the low numbers of participants reporting employment at either 6 or 12 months (25.2%), and the number of employed participants reporting no impact (95.1%) of CVD risk factors on their employment. Participants randomised to usual care reported higher mean per patient private healthcare costs than in the intervention arm in available cases, but lower out-of-pocket expenditure than intervention participants.

These costs were associated with a marginal increase in the proportion of responders (defined in the published trial protocol as the maintenance or reduction of 10-year cardiovascular risk estimated on the basis of the QRISK2 score after 12 months) in the intervention arm, with improvements in EQ-5D-5L utility at the end of 12 months of follow-up, and with a small QALY improvement.

There was a marginal increase in response to treatment (as defined above) in the treatment arm. The intervention was also associated with reductions in blood pressure (mean difference in systolic pressure −2.7 mm Hg; 95% CI −4.7 to −0.6) and in weight (−1.0 kg; 95% CI −1.8 to −0.3). Improvements in diet, physical activity, medication to care and satisfaction with treatment for participants randomised to the intervention arm were also observed. The intervention did not improve cholesterol or smoking status.

### Cost-effectiveness

**Figure 1:**
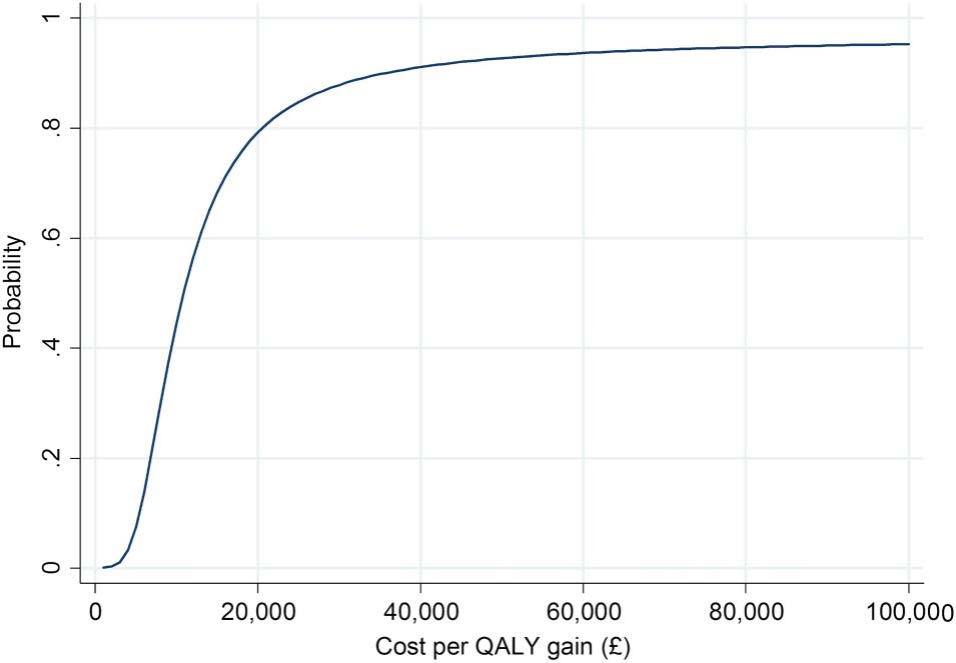
Cost-effectiveness acceptability curve from an NHS perspective for imputed model. NHS, National Health Service; QALY, quality-adjusted life year.

Cost-effectiveness results from an NHS perspective are presented in table 5. The ICER is £10 859 and there is a probability of 0.77 that the intervention is cost-effective at a threshold value of £20 000 per QALY. The probability that the intervention is cost-effective at other values of the cost-effectiveness threshold is shown in figure 1. The between-arm QALY difference of 0.012 corresponds to ∼4 additional days in ‘perfect’ health over the course of a year for participants randomised to the intervention arm rather than to the control arm.

**Table 5:** Cost-effectiveness of the intervention from an NHS perspective

Cost of services	Usual care mean	Intervention mean	Incremental difference (95% CI)
Costs and QALYs			
Total NHS costs	£364	£502	£138 (66 to 211)
Adjusted QALYs	0.786	0.798	0.012 (−0.001 to 0.026)
Cost-effectiveness statistics			
ICER: £10 859			
Probability that intervention cost-effective at CE threshold of £20 000: 0.77			
Probability that intervention cost-effective at CE threshold of £30 000: 0.87			
NMB at threshold of £20 000 (95% CI): £116 (105 to 128)			

CE, cost-effectiveness; ICER, incremental cost-effectiveness ratio; NHS, National Health Service; NMB, net monetary benefit; QALYs, quality-adjusted life years.

### Sensitivity analyses

The base case results are insensitive to the exclusion of secondary NHS costs. The effect of removing these costs is to slightly narrow cost differences between arms (relative to the base case) so that the ICER reduces to £10 003, and hence these costs do not have a material impact on the likely cost-effectiveness of the intervention.

Table 6 presents the results of a complete case analysis of cost-effectiveness from an NHS perspective. The CEAC associated with these results is presented as online supplementary figure A1. The complete case results are similar to those of the imputed base case results.

**Table 6:** Sensitivity analysis: cost-effectiveness complete case from an NHS perspective

Cost of services	Usual care (n=266) mean	Intervention (n=262) mean	Incremental difference (95% CI)
Costs and QALYs			
Total NHS costs—complete case	£367	£490	£124 (42 to 206)
QALYs—complete case	0.788	0.800	0.011 (−0.001 to 0.025)
Cost-effectiveness statistics			
ICER: £10 366			
Probability that intervention cost-effective at CE threshold of £20 000: 0.79			
Probability that intervention cost-effective at CE threshold of £30 000: 0.87			
NMB at threshold of 20 000 (95% CI): £115 (103 to 127)			

CE, cost-effectiveness; ICER, incremental cost-effectiveness ratio; NHS, National Health Service; NMB, net monetary benefit; QALYs, quality-adjusted life years.

## Discussion

We conducted a within-trial evaluation of cost-effectiveness of a de novo telehealth intervention in patients with elevated CVD risk. Trial participants in the control arm received usual care. Participants were followed up for 12 months. Resource use data were collected at 6 and 12 months, and information on health-related quality of life was measured using responses to the EQ-5D-5L questionnaire. Using a health system perspective, we compared healthcare costs to QALYs in the base case cost-effectiveness analysis.

The between-arm difference in QALYs is modest. This small improvement in QALYs in the intervention arm may reflect the indirect impact of engaging with and being supported by the HIAs. For example, intervention participants reported greater medication adherence, better access to support and satisfaction with treatment than did control participants (C Salisbury, A O'Cathain, C Thomas, *et al.* Under review). Intervention participants reported small improvements in blood pressure and BMI, but not with respect to smoking or cholesterol (C Salisbury, A O'Cathain, C Thomas, *et al.* Under review). The balance of these impacts—on the sense of being supported, greater satisfaction with treatment and some improvements in ‘hard’ health indicators such as weight—may have contributed to EQ-5D-5L responses that favoured the intervention.

### Strengths

The Healthlines trial was one of the largest RCTs designed to evaluate a telehealth-based complex intervention for the management of CVD risk. The intervention was based on a programme of work (C Salisbury, A O'Cathain, C Thomas, *et al.* Under review) intended to establish the acceptability of telehealth to patients with chronic conditions, and to support the development of an evidence-based, responsive telehealth service for evaluation in the context of a pragmatic trial design. This economic evaluation contributes to the small body of evidence concerned with the cost-effectiveness of telehealth for patients with long-term conditions.

The economic evaluation was conducted alongside the trial, and prospectively collected detailed patient-level data. The analysis was conducted in line with best practice guidance.^35^ The amount of missing data in the cost and quality of life variables necessary to conduct inferential cost-effectiveness analysis was similar in each arm. Imputed and complete case cost-effectiveness results were similar.

Adherence to the intervention was relatively strong, with a median of 10 out of 13 scheduled encounters received. This suggests that patients randomised to the intervention were willing to engage with the Healthlines Service.

### Limitations

The economic evaluation did not seek to measure the scalability of the intervention to larger (eg, national) patient groups, although the intervention was specifically designed to be easily scaled-up. It is conceivable that scale economies (eg, training more HIAs at the same cost) and other savings (eg, through very large bulk purchases of blood pressure monitors) could be realised to reduce overall intervention cost. There was no evidence from the trial that substantial efficiencies could have been secured but were left unexploited. A related limitation is that the recruitment rate of the trial was relatively low.^11^ This will affect the generalisability of the findings, although it is unclear whether low reluctance is due to lack of interest in telehealth or unwillingness to participate in research.

The 12-month follow-up period of the trial means that questions concerning long-term outcomes cannot be answered definitively, and nor can the issue of whether time-limited (eg, for 12 months) or ongoing (eg, until QRISK2 score was reduced to some target level) telehealth support would be most cost-effective. The intervention may affect the health of participants beyond the end of follow-up for at least two reasons: any improvements in habits and self-management are maintained, and the reduced QRISK2 scores in the intervention during trial follow-up will (slightly) reduce the likelihood of future CVD events occurring. A companion paper^14^ describes the results of a simulation intended to assess the cost-effectiveness of the intervention from a lifetime perspective.

### Other literature

Incremental costs per patient in the intervention arm were £138. There was a small QALY difference between arms of 0.012. We note that Bergmo's^36^ review of QALY gains in telehealth interventions found that 17 included studies reported small, positive effects on quality of life. This is consistent with the results reported here. Overall, the intervention is likely to be cost-effective at conventional NHS cost-effectiveness thresholds.

Comparison of the findings of this economic evaluation with other literature is complicated by differences between studies in technologies assessed, the types of condition analysed, study design and patient population. Evidence for the cost-effectiveness of telehealth in general is mixed,^36–39^ and has been described as being of low quality in some cases.^36^ ^40–42^

A notable example of cost-effectiveness analysis within an English NHS context of telehealth for long-term conditions was evaluation of the ‘Whole Systems Demonstrator’ (WSD) project.^43^ ^44^ Patients with a long-term condition (heart failure, chronic obstructive pulmonary disease or diabetes) received either telehealth (n=845) or usual care (n=728) for 12 months following cluster randomisation of practices in three study sites.

The WSD intervention comprised the use of a broad class of telehealth and telemonitoring equipment, which differed by study site. Participants were asked to take readings using this equipment at the same time each day up to 5 days per week. Specialist nurses monitored and responded to information from patients.

The adjusted estimated QALY gain after 12 months in the WSD study was 0.012 in favour of the intervention, the same mean difference reported here for Healthlines, despite the differences in technology, patient characteristics and study design. NHS and social care costs were substantially higher than in Healthlines, with WSD intervention arm costs £1110 higher than in the control arm. The costs of the intervention itself were estimated to be substantially higher than in Healthlines (£1847 average annual cost for those with equipment and reporting costs at 12-month follow-up in WSD, compared with £129 in Healthlines), although other costs were lower in the intervention arm. The estimated WSD ICER was £92 000.

This high-level comparison between the cost-effectiveness results of the Healthlines trial and from the WSD trial indicate the sensitivity of the cost-effectiveness results to the type and costs of technology evaluated. Despite the same mean QALY difference, the WSD evaluation suggested that substantial changes in intervention cost would be necessary before the service could be considered as a cost-effective means of managing patients with long-term conditions. In contrast to the WSD, the Healthlines intervention was designed to support self-management of chronic diseases at low cost, using readily available low-cost technology such as accessible websites, and telephone support from staff without clinical training.

These considerations are relevant to future study design in this area. Continuing evolution in technology and technology cost is likely to influence both the effectiveness and cost-effectiveness of new interventions.

## Conclusion

The Healthlines RCT provided weak evidence of a modest effect on 10-year CVD risk of the telehealth intervention on the primary binary outcome of response to treatment. However, the intervention was estimated to be cost-effective (measured as a function of the ratio of incremental costs to incremental QALYs) from an NHS perspective after 12 months of trial follow-up.
