# Supplementary material for: Cost-effectiveness of telehealth for patients with raised cardiovascular disease risk: evidence from the Healthlines randomised controlled trial
Source: BMJ Open. 2016 Aug 26;6(8):e012352. doi: 10.1136/bmjopen-2016-012352 (PMC5013404; doi:10.1136/bmjopen-2016-012352)
Supplement: Supplementary data [file bmjopen-2016-012352supp.pdf]

Healthlines CVD 12 month economic evaluation – Online supplementary information

Table A1 Unit costs for primary care and personal social services [a]

Services	Unit Cost	Comments
GP clinic consultation	34.00	Based on a 11.7-minute consultation
GP telephone consultation	20.00	Based on a 7.1-minute consultation
GP home visit	85.00	Based on a 23.4-minute visit
Out-of-hours GP service face-to-face at clinic	25.03	Estimate derived from Scott[1] and increased by 22% as per O'Dowd,[2] inflated to 2012/13 prices
GP out-of-hours service home visits	62.56	Adjusted for duration in line with GP consultations
GP out-of-hours service phone calls	14.72	Adjusted for duration in line with GP consultations
Practice nurse clinic consultation	11.37	Based on 15.5-minute consultation
Practice nurse telephone consultation	6.69	Based on 9.4-minute consultation
**Other primary and community care**		
Counsellor clinic consultation	58.00	Assume 55-minute consultation
Counsellor telephone consultation	15.75	Assume average length of call is 15 minutes
Dietician	14.67	Assume 20-minute consultation
District nurse home visit	60.00	Includes non-contact time related to the visit, assume visits plus these activities take one hour
District nurse phone consultation	10.50	Assume average length of call is 15 minutes
Health trainer	14.67	Same as practice nurse, but assumed appointment time of 20 minutes
Midwife	12.75	Assumed a 15-minute consultation
NHS walk-in centre	34.06	Munro *et al.*,[3] inflated to 2012/13 prices

NHS walk-in centre phone consultation	22.23	Munro *et al.*,[3] inflated to 2012/13 prices
Occupational therapist clinic consultation	34.00	Assume 23-minute consultation
Occupational therapist home visit	62.09	Assume 42-minute visit
Occupational therapist phone consultation	18.72	Assume average length of call is 12.7 minutes
Phlebotomist consultation	6.46	Assume a 15.5-minute consultation
Physician associate	22.00	Costed as per specialist nurses
Physiotherapist	30.00	Assume one-hour consultation
Podiatry	30.00	Assume one-hour consultation
Specialist nurse (e.g. asthma nurse)	22.00	Costed per consultation

a The source of the costs for the services in this table is Curtis,[4] unless otherwise referenced

Table A2 National average unit costs of hospital-related services[a]

Services	National average unit cost (£)
**CVD risk**	
**Inpatient stays**	
Arrhythmia or Conduction Disorders with CC Score 4-6	571
Major Knee Procedures for Non-Trauma, Category 2, without CC	5,676
Minor Vascular Interventional Radiology Procedures	766
Syncope or Collapse with CC Score 0-3	528
Unspecified Chest Pain with CC Score 0-4	533
**Day care and outpatient visits**	
Arrhythmia or Conduction Disorders with CC Score 0-3	818
Cardiology: Non-Admitted Face to Face Attendance, First	167
Cardiology: Non-Admitted Face to Face Attendance, Follow-up	126
Computerised Tomography Scan, one area, no contrast, 19 years and over	104
Diabetics: Non-Admitted Face to Face Attendance, First	189
Diabetics: Non-Admitted Face to Face Attendance, Follow-up	142
Electrocardiogram Monitoring and stress testing (day case)	477
Electrocardiogram Monitoring and stress testing (outpatient)	136
General Medicine: Non-Admitted Face to Face Attendance, Follow-up	102
Nephrology: Non-Admitted Face to Face Attendance, Follow-up	116
Nuclear Medicine, Category 3	196
Ophthalmology: Non-Admitted Face to Face Attendance, Follow-up	80
Percutaneous Standard Ablation, with CC Score 0-2	2,367
Simple Echocardiogram, 19 years and over	77
Unspecified Chest Pain with CC Score 0-4	690
**Accident and emergency**	
Accident and Emergency - Type 01 Non-Admitted	96
**Paramedic/ambulance**	

See and treat or refer	196
See and treat and convey	255

a The cost of these hospital-related services was based on NHS Reference Costs 2012/13[5] and Curtis.[6]

Table A3 Unit costs of resources involved in delivering telehealth intervention

Primary care services	Cost
Basic HIA annual salary (Band 4)	£20,638.00
Oncosts: National insurance and super annuation	£4,827.00
Overheads	£35,417.00
Cost per hour	£23.61
Training cost per HIA per hour	£0.61
Ratio of contact: non-contact time	2:1
Adjusted cost per hour of HIA time	£36.34
BP monitor	£37.50

HIA = Health Information Advisor; BP = blood pressure.

Table A4 Mean (SD) number of primary care consultations in the depression trial: all available data and complete cases

	Usual care		Intervention	
**Primary care services**	**N**	**Mean (SD)**	**N**	**Intervention mean (SD)**
**All available data**				
GP consultations	313	5.30 (4.53)	325	5.48 (4.38)
Nurse consultations	313	3.03 (4.31)	325	2.79 (2.93)
Other primary care consultations	313	3.09 (3.91)	325	3.01 (3.95)
**Complete cases**				
GP consultations	266	5.21 (4.42)	262	5.09 (3.79)
Nurse consultations	266	3.06 (4.50)	262	2.83 (3.05)
Other primary care consultations	266	3.14 (3.87)	262	3.05 (4.18)

Table A5 Mean (SD) cost (£) per participant of primary care consultations in the trial: all available data and complete cases

	Usual care		Intervention	
**Primary care services**	**N**	**Mean £ (SD)**	**N**	**Mean £ (SD)**
**All available data**				
GP consultations	313	168.92 (143.25)	325	172.48 (131.77)
Nurse consultations	313	33.82 (48.34)	325	31.12 (32.68)
Other primary care consultations	313	19.14 (26.68)	325	19.13 (26.00)
Total primary care cost	313	221.88 (166.20)	325	222.73 (150.70)
**Complete cases**				
GP consultations	266	165.46 (137.19)	262	160.74 (116.66)
Nurse consultations	266	34.27 (50.53)	262	31.66 (34.06)
Other primary care consultations	266	19.27 (25.94)	262	19.35 (27.45)
Total primary care cost	266	219.00 (164.17)	262	211.76 (138.99)

Table A6 Mean (SD) number of prescribed medication items and cost (£) per participant in the depression trial: all available data and complete cases

	Usual care		Intervention	
**Primary care services**	**N**	**Mean (SD)**	**N**	**Mean (SD)**
**No. of prescribed items**				
All available data	313	20.83 (21.41)	325	20.52 (20.79)
Complete cases	266	21.08 (21.40)	262	20.91 (21.33)
**Cost of prescribed medication (£)**				
All available data	313	£67.38 (136.56)	325	£66.59 (102.38)
Complete cases	266	£67.78 (139.04)	262	£65.21 (102.51)

Table A7 Mean (SD) number of contacts with NHS community services per participant in the trial: all available data and complete cases

	Usual care		Intervention	
**Community services**	**N**	**Mean(SD) number of contacts**	**N**	**Mean (SD) number of contacts**
**All available data**				
District nurse home visit	290	0.07 (0.52)	292	0.08 (1.18)

District nurse phone consultation	290	0.01 (0.08)	292	0.09 (0.66)
NHS counsellor/psychologist clinic visit	290	0.08 (0.61)	292	0.10 (0.81)
NHS counsellor/psychologist phone consultation	290	-	292	0.16 (0.92)
NHS walk-in centre clinic visit	290	0.21 (1.09)	292	0.17 (1.33)
NHS walk-in centre phone consultation	290	0.02 (0.23)	292	0.07 (0.69)
GP out-of-hours service clinic visit	290	0.03 (0.25)	292	0.05 (0.36)
GP out-of-hours service home visit	290	0.01 (0.08)	292	0.01 (0.13)
GP out-of-hours service phone consultation	290	0.05 (0.35)	292	0.11 (0.70)
**Complete cases**				
District nurse home visit	266	0.05 (0.45)	262	0.01 (0.12)
District nurse phone consultation	266	0.00 (0.06)	262	0.10 (0.69)
NHS counsellor/psychologist clinic visit	266	0.08 (0.63)	262	0.08 (0.77)
NHS counsellor/psychologist phone consultation	266	-	262	0.18 (0.97)
NHS walk-in centre clinic visit	266	0.23 (1.13)	262	0.16 (1.32)
NHS walk-in centre phone consultation	266	0.03 (0.24)	262	0.06 (0.72)
GP out-of-hours service clinic visit	266	0.03 (0.27)	262	0.04 (0.29)
GP out-of-hours service home visit	266	0.01 (0.09)	262	0.01 (0.12)
GP out-of-hours service phone consultation	266	0.05 (0.36)	262	0.10 (0.72)

Table A8 Mean (SD) cost (£) per participant of NHS community services in the trial: all available data and complete cases

	**Usual care**		**Intervention**	
**Primary care services**	**N**	**Mean £ (SD)**	**N**	**Mean £ (SD)**
**All available data**				
District nurse home visit	285	4.21 (31.56)	285	4.63 (71.41)
District nurse phone consultation	285	0.07 (0.88)	285	0.96 (6.98)
NHS counsellor/psychologist clinic visit	285	4.48 (35.48)	285	5.90 (47.45)

NHS counsellor/psychologist phone consultation	285	-	285	2.65 (14.72)
NHS walk-in centre clinic visit	285	7.29 (37.37)	285	5.98 (45.88)
NHS walk-in centre phone consultation	285	0.55 (5.08)	285	1.33 (15.38)
GP out-of-hours service clinic visit	285	1.07 (8.73)	285	1.67 (12.32)
GP out-of-hours service home visit	285	0.60 (7.11)	285	0.89 (11.24)
GP out-of-hours service phone consultation	285	0.91 (6.96)	285	2.18 (14.22)
Total costs for available cases	285	19.18 (63.69)	285	26.19 (117.03)
**Complete cases**				
District nurse home visit	266	3.16 (26.90)	262	0.46 (7.41)
District nurse phone consultation	266	0.04 (0.64)	262	1.04 (7.28)
NHS counsellor/psychologist clinic visit	266	4.80 (36.71)	262	4.87 (44.57)
NHS counsellor/psychologist phone consultation	266	-	262	2.83 (15.31)
NHS walk-in centre clinic visit	266	7.68 (38.61)	262	5.46 (44.89)
NHS walk-in centre phone consultation	266	0.59 (5.26)	262	1.44 (16.04)
GP out-of-hours service clinic visit	266	1.15 (9.03)	262	1.30 (9.79)
GP out-of-hours service home visit	266	0.64 (7.36)	262	0.65 (10.50)
GP out-of-hours service phone consultation	266	0.98 (7.20)	262	2.06 (14.34)
Total costs for complete cases	266	19.03 (63.38)	262	20.11 (93.07)

Table A9 Mean (SD) number of hospital encounters and use of ambulance services in the trial: all available data and complete cases

	Usual care		Intervention	
**Services**	**N**	**Mean (SD) number of encounters**	**N**	**Mean (SD) number of encounters**
**All available data**				
Overnight hospital stay	296	0.01 (0.10)	297	0.01 (0.12)
Day care	296	0.03 (0.18)	297	0.01 (0.08)
Outpatient clinic	293	0.04 (0.33)	294	0.04 (0.26)
Accident and emergency	298	0.01 (0.10)	299	0.01 (0.14)

Other hospital services	297	0.01 (0.12)	299	0.01 (0.10)
Ambulance use	294	0.01 (0.08)	296	0.01 (0.10)
**Complete cases**				
Overnight hospital stay	266	0.01 (0.11)	262	0.02 (0.12)
Day care	266	0.03 (0.19)	262	0.01 (0.09)
Outpatient clinic	266	0.04 (0.35)	262	0.04 (0.27)
Accident and emergency	266	0.01 (0.11)	262	0.02 (0.15)
Other hospital services	266	0.02 (0.12)	262	0.01 (0.09)
Ambulance use	266	0.01 (0.09)	262	0.01 (0.11)

Table A10 Mean (SD) cost (£) per participant of hospital and ambulance care in the trial: all available data and complete cases

	**Usual care**		**Intervention**	
**Services**	**N**	**Mean £ (SD)**	**N**	**Mean £ (SD)**
**All available data**				
Overnight hospital stay	296	6.31 (63.35)	297	24.73 (333.73)
Day care	298	15.68 (153.05)	299	1.08 (13.46)
Outpatient clinic	293	11.44 (143.49)	295	4.82 (30.34)
Accident and emergency	298	0.64 (7.83)	299	1.28 (13.53)
Other hospital services	297	1.12 (13.82)	299	1.31 (15.98)
Ambulance use	294	1.73 (21.00)	296	2.58 (25.58)
Mean cost for available cases[a]	283	38.61 (346.27)	293	37.55 (352.79)
**Complete cases**				
Overnight hospital stay	266	7.03 (66.80)	262	28.03 (355.27)
Day care	266	17.56 (161.92)	262	1.23 (14.37)
Outpatient clinic	266	12.60 (150.57)	262	4.94 (31.27)
Accident and emergency	266	0.72 (8.29)	262	1.46 (14.45)
Other hospital services	266	1.25 (14.60)	262	0.75 (12.09)

Ambulance use	266	1.92 (22.07)	262	2.92 (27.18)
Mean cost for complete cases	266	41.08 (357.06)	262	39.34 (366.13)

a The mean cost for all available cases is summed over available cases across all categories of care reported, and hence is not equal to the sum of individual costs.

Figure A1 Cost-effectiveness acceptability curve for complete case analysis

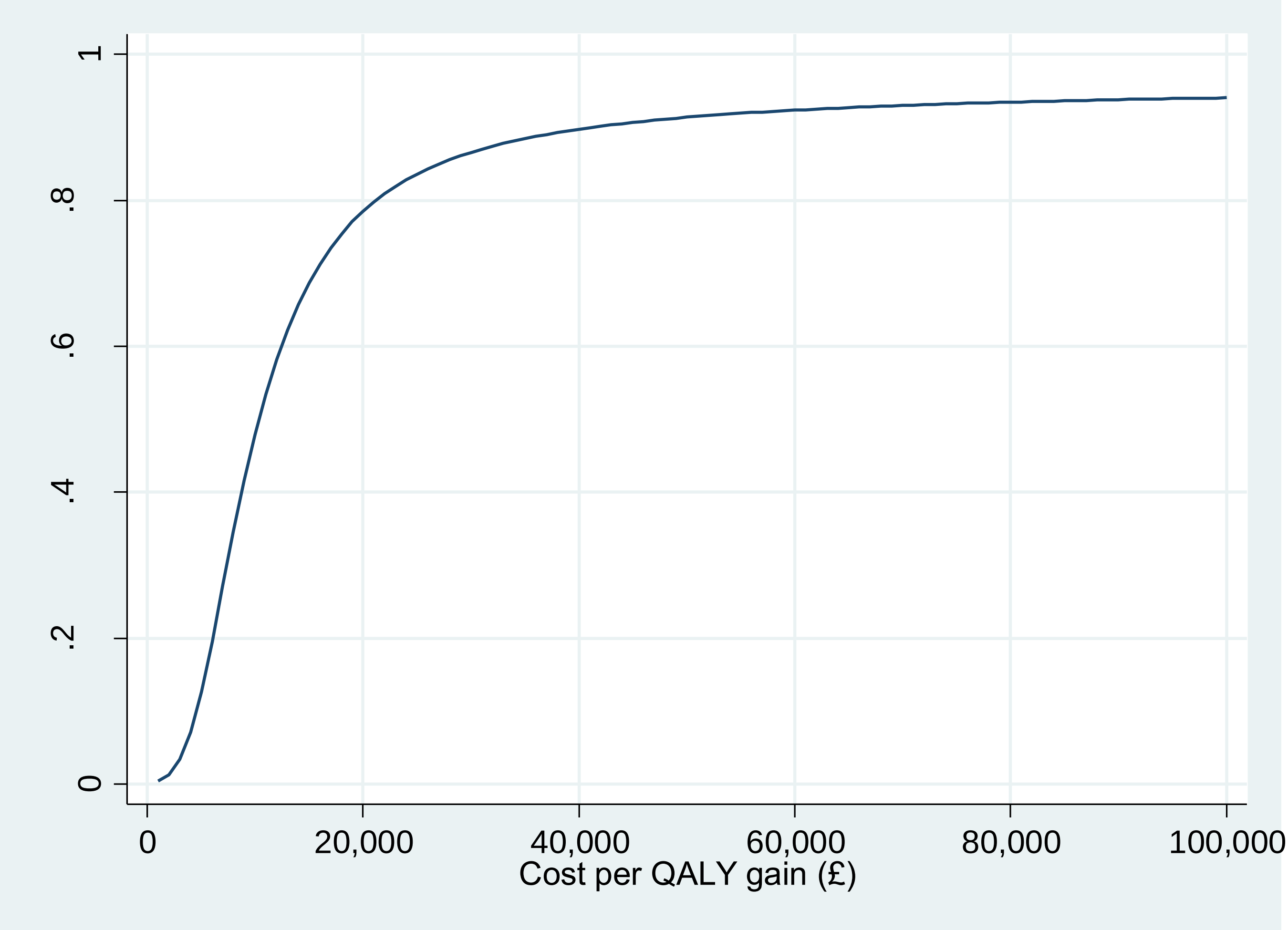

References

1. Scott A, Simoens S, Heaney D, et al. What does GP out of hours care cost? An analysis of different models of out of hours care in Scotland. ScottMed J 2004;**49**(2):61-66.
2. O'Dowd A. Cost of out of hours care was 22% higher than predicted in England. BMJ 2006;**332**(7550):1113.
3. Munro J, Nicholl J, O'Cathain A, et al. Evaluation of NHS Direct first wave sites: Final report of the phase 1 research. Sheffield: MCRU University of Sheffield, 2001:1-84.
4. Curtis L. Unit Costs of Health and Social Care. Canterbury: Personal Social Services Research Unit, 2013.

5. Department of Health. Reference Costs 2012/13. London, 2013.
6. Curtis L. *Unit Costs of Health and Social Care 2013*. Canterbury: Personal Social Services Research Unit, 2013.
